# Grindelia Robusta in Asthma and Chronic Bronchitis

**Published:** 1893-07-15

**Authors:** 


### NEW DRUGS AND PREPARATIONS.

[We should be glad to receive, at our office, 428, Strand, London, W.C., from the manufacturers, specimens of all new preparations and drugs which may be brought out from time to time.]

### GRINDELIA ROBUSTA IN ASTHMA AND CHRONIC BRONCHITIS.

The drug which is the subject of this note, though by no means very recently introduced, is, nevertheless, comparatively little known, yet the extremely good results which sometimes follow its administration in cases which are proverbially some of the most capricious and obstinate with which we have to deal entitle it to a wider reputation and more frequent use. Like all other remedies for asthma, this drug entirely fails to relieve a large number of cases, but in those which are benefited by its action the effect is often very striking.

Grindelia robusta, known as the Gum Plant, and first employed medicinally in America, is not contained in the British Pharmacopœia. It is, however, included in the Extra Pharmacopœia of Messrs. Martindale and Westcott, and is favourably mentioned in Dr. Ringer's "Handbook of Therapeutics." There are two preparations, namely, the extract and liquid extract, the latter being that with which the writer is alone acquainted. It is a thick yellowish-green liquid, which, when suspended in water by the aid of mucilage—simple admixture causing precipitation of the resin—forms an opaque green preparation of very unattractive appearance. It has an extremely strong resinous and decidedly offensive taste, which it is quite useless to attempt to mask, and causes an unpleasant burning sensation at the back of the throat. Indeed, it must be admitted that it has nothing but its effect to recommend it, and were the symptoms for which it is used less distressing it is probable that considerable objections would be raised by patients to its administration.

The liquid extract is often prescribed in ʒss doses, to be taken regularly three or four times a day; but in cases of true spasmodic asthma it is better to give it either shortly before the onset of, or during a paroxysm. The writer has found that the best results ensue in these cases from the administration of a dose an hour or so before the time at which the attack usually begins, such attacks generally following, with considerable precision, a fairly definite course as regards their time of onset. Should the paroxysm fail to be averted by this means, another dose should then be given, and repeated at intervals of one or two hours during its continuance. For such purposes the first two doses should amount to as much as ʒj each, whilst, afterwards, it will be advisable to diminish the quantity taken to ʒss, or even ♏xx, according to the effect produced. When given in this manner the drug not infrequently affords very great relief to the symptoms, and the writer has seen one very striking instance in which complete cessation of attacks of a very severe and obstinate nature speedily followed its employment. Grindelia is also often of service in the treatment of cases of chronic bronchitis, especially those in which definite asthmatic attacks occur at intervals, and also those in which the dyspnœa, though not paroxysmal, resembles in its character that associated with asthma, that is, where there is a sense of great inspiratory effort, with little or no bronchial secretion. In such cases the addition of ʒss of the liquid extract to the medicines ordinarily in use for this condition will often afford very great relief. It must, however, be remembered that grindelia is a drug the efficacy of which becomes very speedily lost by frequent repetition; and patients who are benefited by its use cannot be too strongly advised to keep it in reserve as far as possible, and, at any rate, to avoid taking it continuously for any considerable length of time.

